# Nanopore and Illumina sequencing reveal different viral populations from human gut samples

**DOI:** 10.1099/mgen.0.001236

**Published:** 2024-04-29

**Authors:** Ryan Cook, Andrea Telatin, Shen-Yuan Hsieh, Fiona Newberry, Mohammad A. Tariq, Dave J. Baker, Simon R. Carding, Evelien M. Adriaenssens

**Affiliations:** 1 Quadram Institute Bioscience, Norwich, NR4 7UQ, UK; 2 Department of Biosciences, Nottingham Trent University, Nottingham, NG11 8NS, UK; 3 Faculty of Health and Life Sciences, University of Northumbria, Newcastle upon Tyne, NE1 8ST, UK; 4 Norwich Medical School, University of East Anglia, Norwich, NR4 7TJ, UK

**Keywords:** human virome, Illumina, microbiome, Nanopore, viromics

## Abstract

The advent of viral metagenomics, or viromics, has improved our knowledge and understanding of global viral diversity. High-throughput sequencing technologies enable explorations of the ecological roles, contributions to host metabolism, and the influence of viruses in various environments, including the human intestinal microbiome. However, bacterial metagenomic studies frequently have the advantage. The adoption of advanced technologies like long-read sequencing has the potential to be transformative in refining viromics and metagenomics. Here, we examined the effectiveness of long-read and hybrid sequencing by comparing Illumina short-read and Oxford Nanopore Technology (ONT) long-read sequencing technologies and different assembly strategies on recovering viral genomes from human faecal samples. Our findings showed that if a single sequencing technology is to be chosen for virome analysis, Illumina is preferable due to its superior ability to recover fully resolved viral genomes and minimise erroneous genomes. While ONT assemblies were effective in recovering viral diversity, the challenges related to input requirements and the necessity for amplification made it less ideal as a standalone solution. However, using a combined, hybrid approach enabled a more authentic representation of viral diversity to be obtained within samples.

**Impact Statement**

Viral metagenomics, or viromics, has revolutionised our understanding of global viral diversity, although long-read and hybrid approaches are not yet widespread in this field. Here, we compared the performance of Illumina short-read and Nanopore long-read assembly approaches for recovering fully resolved viral genomes from human faecal samples. We show that Illumina's short-read sequencing is superior for recovering fully resolved viral genomes, while Oxford Nanopore Technology's long-read sequencing is superior in capturing broader viral diversity. However, a hybrid approach, utilising both technologies, may mitigate the limitations of one technology alone.

## Data Summary

All reads used in this study are available on European Nucleotide Archive (ENA) within the project PRJEB47625. The assemblies are available on Zenodo via https://zenodo.org/records/10650983.

## Introduction

The study of uncultivated viruses through viral metagenomics, hereafter viromics, has shaped our understanding of global viral diversity. It is now more than 20 years since the sequencing of the first virome with the revolutionary linker-amplified shotgun library (LASL) approach [1]. Since then, the advent of high-throughput sequencing has driven a viromics revolution.

Viromics has uncovered ecological roles of viruses in diverse environments, shed light on the contribution of bacteriophages (hereafter phages) to the metabolism of their hosts [2,4], uncovered phages as key players in the human intestinal microbiome [5,6], and allowed for the construction of uncultivated genomes larger than any of those obtained from culturing [7,8], which are now being used to inform taxonomy [9,12]. However, methodological developments within the field of bacterial metagenomics are yet to be widely adopted within viromics. Bacterial metagenomics has taken advantage of long-read sequencing technology, most notably Oxford Nanopore Technologies (ONT) and Pacific Bioscience (PacBio) to study complex ecosystems and microbiomes. Long-read and hybrid assemblies have advanced bacterial genomics and metagenomics, improving the completeness of metagenome-assembled genomes (MAGs) and in some cases identifying modified DNA [13,18]. Although phages, the most abundant viruses in a virome, have far smaller genomes than their hosts, they are not always easily resolved with short-read sequencing alone. For example, phages of the ubiquitous bacterial order Candidatus Pelagibacterales possess genomes with high micro-diversity and/or genomic islands [19,20], which are known to cause fragmentation during assembly [21,24]. Long reads have the potential to cover the entire length of a viral genome, thus offering a solution to these issues.

There are a few notable examples of long-read and hybrid sequencing approaches being used for viromics. A pooled and multiple displacement amplification (MDA)-amplified PromethION library was used alongside non-amplified Illumina libraries in a study of agricultural slurry, with the addition of ONT reads increasing the recovery of viral genomes [25]. Similarly, a long-read LASL approach sequenced on a MinION and paired with Illumina sequencing, dubbed VirION and later improved for VirION2, has also increased the number and completeness of viral genomes from marine samples [26,27]. Furthermore, the inclusion of ONT reads has increased the recovery of viral genomes from groundwater metagenomes [28]. Additionally, a hybrid assembly approach has improved the assembly completeness and quality of individual phage genomes belonging to the genus *Przondovirus* [29], utilising similar methods to those suggested for high-quality bacterial genome assemblies [30]. However, whilst long-read sequencing may aid in the recovery of viral genomes, it is not without limitations and challenges.

Despite continuous improvements as new library preparation kits and flow cells are developed, ONT sequencing has a higher error rate compared to Illumina [31,33]. These higher error rates impact on protein prediction through the introduction of erroneous stop codons, leading to truncated proteins and inaccurate functional annotations [32]. This has been shown to impact on viral identification when protein annotations are used for prediction of viral genomes (e.g. VIBRANT) [34,35]. Polishing ONT virome assemblies with corresponding Illumina libraries can reduce this error rate and increase predicted protein lengths, although still not to the levels of Illumina-only assemblies [25,35]. Furthermore, the yields of DNA obtained from virome extractions are typically low and the large input requirements of ONT sequencing can be prohibitive for samples from some environments (typically 1 µg of DNA in 50 µl of buffer). To overcome the input requirements, one of the first ONT virome studies utilised tangential flow filtration to concentrate large volumes of seawater [36], while others have developed a LASL approach [26,27], and MDA [25,35]. However, MDA introduces biases into metagenomic libraries that can lead to the over-representation of viruses with small, circular ssDNA genomes [37,39]. Previously, the issues of MDA were overcome by pairing MDA-amplified ONT libraries with corresponding non-amplified Illumina libraries [25]. As the strengths and limitations of ONT sequencing are the opposite of those for Illumina, a hybrid approach may allow for the mitigation of both platforms’ limitations.

Whilst there have been limited comparisons of long- and short-read sequencing for viromes, a recent study benchmarked sequencing technologies and assembly algorithms using a mock community of 15 phage genomes [35]. This work concluded that, as a single technology, Illumina performed best at recovering complete viral genomes [35]. However, the addition of long reads (particularly ONT) increased recovery of viral genomes [35]. Here, we sought to investigate the impact of sequencing platform and assembly strategy on the recovery of viral genomes from human faecal samples and to offer recommendations for virome analyses.

## Methods

### Sample collection, processing, and sequencing

Sample collection, virome preparation, and Illumina sequencing were performed as part of a previously described comparison of PCR versus PCR-Free DNA library preparation for human faecal viromes [40]. The ethics for the previous study were approved by the University of East Anglia (UEA) Faculty of Medicine and Health Sciences (FMH) Research Ethics Committee (FMH20142015-28), Norwich in 2014, and by the Health Research Authority (HRA) NRES Committee (17/LO/1102; IRAS: 218545) in 2017. In brief, three human faecal samples were collected from healthy adult male donors. Samples were homogenised in sterile TBT buffer, centrifuged at 11 200 ***g*** for 30 min at 10 °C for two rounds, and filtered sequentially through 0.8 and 0.45 µm PES cartridge filters. Filtrates were PEG-precipitated to concentrate virus-like particles (VLPs), followed by treatment with DNAse and RNAse to remove free nucleic acids. DNA was extracted following a standard phenol : chloroform : isoamyl alcohol protocol. To gain enough material for ONT sequencing, multiple DNA extracts were performed on aliquots of the same sample and then pooled. For full details, please see the previous publication [40].

Illumina libraries were sequenced using 2×150 bp paired-end chemistry (PE150) on the Illumina HiSeq X Ten platform [40]. This study uses the PCR-free Illumina libraries described in the previous publication [40]. ONT libraries were prepared using the SQK-LSK109 kit and sequenced on a MinION with r9.4.1 flow cells. ONT basecalling was performed with Guppy v6.4.6 using model dna_r9.4.1_450bps_ha.

### Quality control of reads

Illumina reads were trimmed with BBTools v39.01 following a previously reported protocol [41]. Reads were initially trimmed of adapters with bbduk.sh using `ktrim=r minlen=40 minlenfraction=0.6 mink=11 tbo tpe k=23 hdist=1 hdist2=1 ftm=5 ref=adapters.fa`, followed by quality trimming with bbduk.sh using `maq=8 maxns=1 minlen=40 minlenfraction=0.6 k=27 hdist=1 trimq=12 qtrim=rl`, and error corrected with tadpole.sh using `mode=correct ecc=t prefilter=2` (https://jgi.doe.gov/data-and-tools/software-tools/bbtools/). ONT reads were filtered using Filtlong v0.2.1 using `--min_length 500 --keep_percent 90` (https://github.com/rrwick/Filtlong). Reads were inspected before and after trimming with FastQC v0.11.8 for Illumina reads (https://www.bioinformatics.babraham.ac.uk/projects/fastqc/), and NanoPlot v1.41.6 for basecalled ONT reads [42]. Read length distributions were summarised using the `stats` command as part of SeqFu v1.17.1 [43]. For all assembly combinations, four assemblies were attempted; one for each of the three libraries and an additional co-assembly which pooled the individual libraries.

### Short-read and binning assemblies

Illumina reads were assembled using MEGAHIT v1.2.9 with `--k-min 21 --k-max 149 --k-step 24 --min-contig-len 1500` [44]. Three virus-specific binning approaches were used on each MEGAHIT assembly. (1) vRhyme v1.1.0 was used with fastq files as input using Bowtie 2 v2.5.1 for read mapping [45], and the `--keep_bam` flag was used to retain BAM files for use in another binning tool VAMB [46]. (2) VAMB v4.1.1 was used with default parameters [47]. (3) PHABLES v0.2.0 was used with default parameters [48]. For the ‘pooled’ vRhyme and VAMB assemblies, the contigs were taken from the co-assembly but individual libraries were used for reads/BAMs. For downward processing, bins were concatenated into single contigs and fused with a single N using fuse.sh from BBTools v39.01 (https://jgi.doe.gov/data-and-tools/software-tools/bbtools/). All ‘binning’ assemblies were combined with Illumina contigs native from their respective MEGAHIT assembly.

### Long-read and hybrid assemblies

ONT reads were assembled using a variety of assemblers; all assemblies were performed separately on ‘raw’ reads and those processed with Filtlong. Canu v2.2 was used with `corMinCoverage=0 corOutCoverage=all corMhapSensitivity=high correctedErrorRate=0.105 genomeSize=5 m corMaxEvidenceCoverageLocal=10 corMaxEvidenceCoverageGlobal=10 oeaMemory=32 redMemory=32 batMemory=200` [49]. Flye v2.9.2-b1786 was used with `--nano-hq –meta` [16]. Redbean (wtdbg2) v2.5 was used with `-p 21 k 0 -AS 4 K 0.05 s 0.05 L 1000 --edge-min 2 --rescue-low-cov-edges` [50]. Raven v1.8.1 was used with default parameters [51]. Unicycler v0.5.0 (using miniasm and Racon) was used with default parameters with ONT reads only, as well as hybrid assemblies that used both Illumina and ONT reads from their respective libraries [52,54].

All ONT assemblies underwent four rounds of polishing with Medaka v1.7.3 with model r941_min_hac_g507, which used minimap2 v2.24-r1122 for alignments [55] (https://github.com/nanoporetech/medaka). To produce additional short-read polished assemblies, all medaka polished assemblies underwent one round of polishing with Polypolish v0.5.0 as described in the Polypolish documentation (https://github.com/rrwick/Polypolish). Firstly, reads from respective Illumina libraries were mapped using bwa mem v0.7.17-r1188 [56]. Alignments were filtered using polypolish_insert_filter.py, and Polypolish was then used with default parameters [57].

### Viral identification and ORF prediction

Viruses were predicted from the assemblies using geNomad v1.5.2 [58]. Predicted viruses were subsequently processed using CheckV v1.0.1 that uses db v1.5 [59]. Open reading frames (ORFs) were predicted using Prodigal-gv v2.11.0-gv, a fork of Prodigal [60] that has been optimised for viral gene prediction (https://github.com/apcamargo/prodigal-gv) [58,61].

### Comparison of predicted complete Phables viruses to ONT counterparts

The 81 predicted complete genomes from the pooled Phables assembly were extracted and compared to pooled library ONT assemblies using MASH v2.3 [62]. Highly similar ONT contigs were extracted using a distance cutoff ≤0.05. The Phables predicted complete genomes and highly similar ONT contigs were used as input for Mashtree v1.4.6 with `--reps 100` and `--min-depth 0` [63].

### Recovery of viral diversity

Predicted viruses with a CheckV completeness estimate ≥50 % (medium quality and above) were dereplicated to form viral operational taxonomic units (vOTUs) following Minimum Information about an Uncultivated Virus Genome (MIUViG) standards (95 % ANI over 85 % length) using blast v2.14.0+ alongside the anicalc.py and aniclust.py scripts described in the CheckV documentation (https://bitbucket.org/berkeleylab/checkv/src/master/) [59,64, 65]. Translated proteins predicted using Prodigal-gv v2.11.0-gv were used as input for vConTACT2 v0.11.3 alongside the INPHARED database (July 2023) with `--min-size 1` [66,67]. To assign taxonomy, the vConTACT2 output was processed using graphanalyzer (https://github.com/lazzarigioele/graphanalyzer) [68]. Viral clusters (VCs) were treated as genera, with those containing no reference sequences being described as novel. Pooled Illumina reads were mapped to sequences for which the VC contained ONT-assembled sequences but no Illumina-based assemblies using bbmap.sh BBTools v39.01 (https://jgi.doe.gov/data-and-tools/software-tools/bbtools/), to determine if the VC could be detected in the Illumina data without being assembled. Presence was defined as ≥1× coverage over ≥75 % of contig length [24].

### CrAssphage analysis

Sequences that clustered with CrAssphage LMMB (MT006214) were processed using Clinker v0.0.28 to compare genome synteny and completeness [69].

### Data visualisation

Unless otherwise stated, all plots were produced in R v4.2.2 [70] using ggplot2 v3.4.2 [71]. The vConTACT2 network was visualised using Cytoscape v3.9.1 [72]. Tthe upset plot was produced using UpSetR v1.4.0 [73], and the genome architecture comparison was produced using Clinker v0.0.28 [69]. Fig. S1 (available in the online version of this article) was visualised using IToL [74].

## Results

To compare the performance of commonly used assembly algorithms for recovery of viral genomes from faecal samples, we sequenced three human faecal viromes using Illumina and ONT sequencing and tested several assembly strategies for reconstructing virus genomes from the read datasets (Table S1).

### Data generation

Illumina sequencing of non-amplified viromes was carried out on the HiSeq X Ten platform using 150 bp paired-end libraries. The Illumina libraries generated 5.59, 4.96 and 6.27 Gbp of data, with a mean phred score ≥30 ranging from 92.37–94.35 % (Table S2). Post-trimming and quality control, Illumina libraries were 5.48, 4.9 and 6.16 Gbp, with a mean phred score ≥30 ranging from 92.64–95.73 % (Table S2). To generate enough material for ONT sequencing, multiple DNA extracts from aliquots of the same sample were pooled prior to sequencing on a MinION with r9.4.1 flow cells. ONT libraries generated 8.68, 5.1 and 5.43 Gbp data with median Q scores of 14.1, 13.1 and 13.3, respectively (Table S2). Post-filtering with Filtlong, ONT libraries were 7.73, 4.1 and 3.89 Gbp, with median Q scores of 14.8, 13.4 and 13.9, respectively (Table S2). Summaries of read length distributions for all libraries are shown in Table S2.

### Virome assembly

Assemblies were produced using a variety of strategies and assemblers, including hybrid and binning approaches (Table S1). For each assembler combination, four assemblies were attempted, one for each library and an additional pooled library. Short-read assemblies were performed with MEGAHIT only, as assembly comparisons for Illumina reads have previously shown it to provide high-quality assemblies [24,75]. All ONT assemblies were attempted using both ‘raw’ reads as well as those filtered with Filtlong. Additionally, all ONT assemblies were polished with Illumina reads from their respective library using PolyPolish.

Out of a possible 104 assemblies, 93 were produced successfully (Table S1). Sample 03 failed to assemble using VAMB due to not satisfying the minimum number of contigs required for input (≥4096). Unicycler failed to yield assemblies for sample 01 and the pooled sample when using ONT reads only, and the pooled sample when using ONT and Illumina reads together. The Unicycler assemblies failed both before and after filtering ONT reads with Filtlong. All assemblies were given a maximum of 88 cores and 1500 Gb of memory on an Intel Xeon Gold 6238 CPU @ 2.10 GHz node with 4 CPUs that have 22 cores each; the failure is therefore unlikely to have been due to limited computational resources. However, as Unicycler was designed for single genomes rather than mixed community samples, this was not surprising.

Regarding total assembly size, the largest assemblies were obtained from Illumina data (20–125 Mb per assembly; Tables 1 and S3). However, these assemblies were highly fragmented, containing the highest number of contigs (1851–27 614 per assembly), with the shortest contig lengths (1.85–2.51 Kb median contig lengths per assembly). The total assembly size and median contig length for ONT-based assemblies varied greatly with the assembler used. Unicycler and Raven obtained the highest median contig lengths (22.42–36.45 Kb median contig lengths per assembly), although this was at the cost of smaller total assemblies (7.97–49.4 Mb per assembly). Flye and wtdbg2 produced larger total assemblies (14.86–108.51 Mb per assembly), although with shorter median contig lengths (5.31–11.55 Kb median contig lengths per assembly). Unicycler with ONT and Illumina reads together, and Canu produced small assemblies (7.41–27.72 Mb per assembly) with relatively short contig lengths (6.57–20.1 Kb median contig lengths per assembly). The use of Filtlong prior to assembly had substantial impacts on some ONT assemblers, increasing median contig lengths from 26 to 32.5 Kb and 25.5 to 27 Kb for Unicycler (with ONT reads only) and Raven, respectively. The same effects of Filtlong were not observed for Canu, Flye, wtdbg2, and Unicycler (with ONT and Illumina reads together). Unsurprisingly, polishing ONT assemblies with Illumina reads led to modest reductions in total assembly size.

**Table 1.** Summary statistics of virome contigs and contig bins by assembly type

Type	Min. length (Kb)	Max. length (Kb)	Median (Kb)	Mean (Kb)	sd (Kb)	Min no. of contigs	Max no. of contigs	Median no. of contigs	Mean no. of contigs	sd	Min. assembly (Mb)	Max. assembly (Mb)	Median assembly (Mb)	Mean assembly (Mb)	sd (Mb)
Bins	1.5	2568.45	2.25	6.51	33.44	1851	27 595	9443	10 539.27	7001.78	20.15	124.97	69.75	68.62	40.02
Hybrid	1.5	688.79	7.44	20.57	42.03	352	1460	597	802.67	519.64	9.57	27.72	12.26	16.51	8.75
Illumina	1.5	385.38	2.42	4.62	9.46	3559	27 617	12 319.5	13 953.75	10 188.24	20.15	124.61	56.65	64.51	44.9
ONT	1.5	1301.1	10.45	23.62	47.41	166	6154	694.5	1399.42	1502.44	7.41	108.51	20.88	33.05	27.16
Polished	1.5	1300.91	10.45	23.61	47.4	166	6154	694.5	1399.42	1502.44	7.41	108.48	20.88	33.04	27.15

### Recovery of viral genomes

To compare the performance of the assemblies in recovering viral genomes, we predicted viruses with geNomad and assessed their completeness with CheckV, including genomes with an estimated completeness of ≥50 % (medium quality and above).

**Fig. 1.**
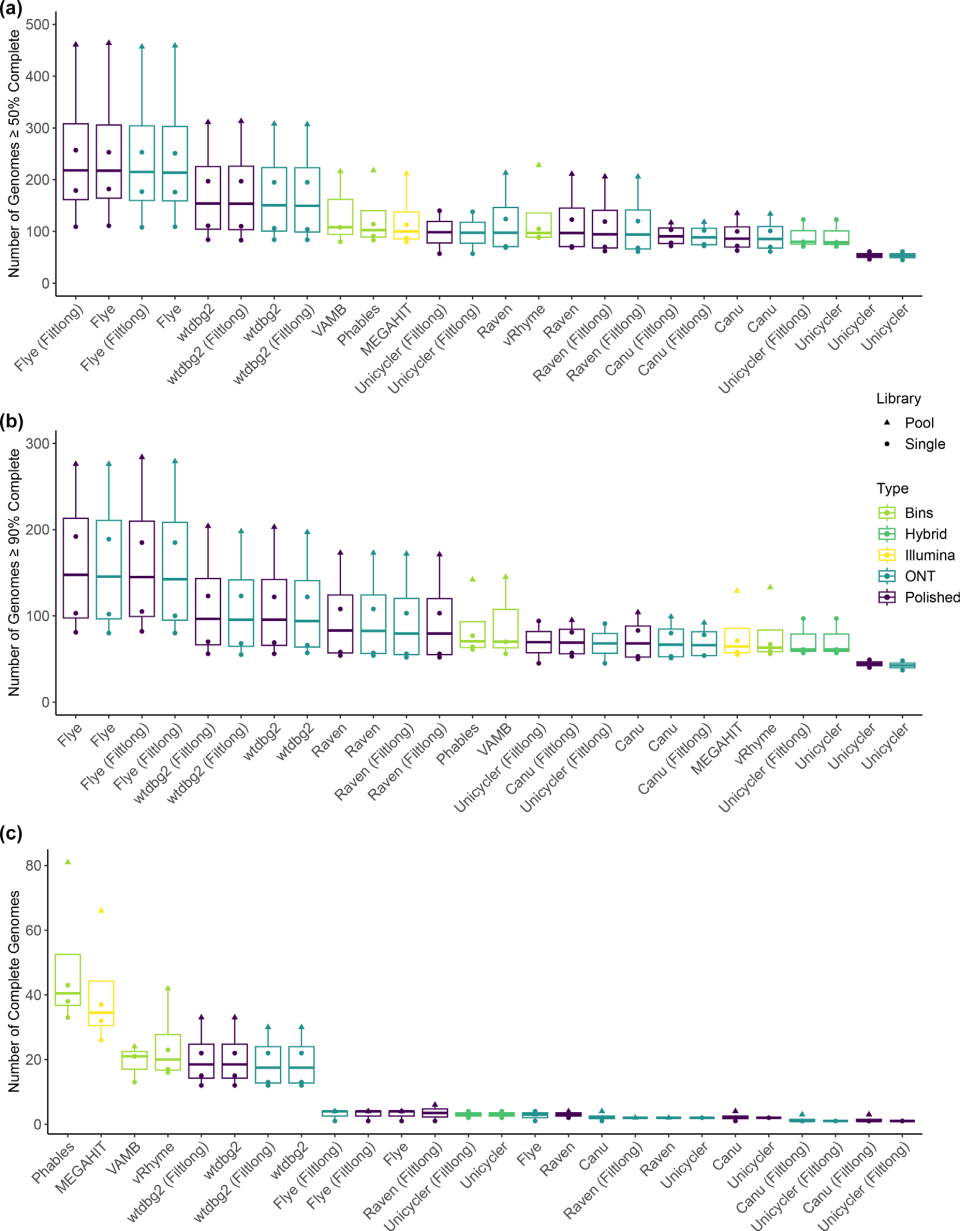
Recovery of viral genomes. The number of viral genomes per assembly predicted to be (**a**) ≥50 % complete, (**b**) ≥90 % complete, and (**c**) 100 % complete. Assemblies are sorted by median value in descending order, showing the best performance on the left-hand side of the plot.

The choice of assembly algorithm used to assemble ONT reads had a substantial effect, as ONT assemblies performed both highest and lowest for viral genome recovery depending on the assembler used (Fig. 1a and Table S4). The Flye assemblies obtained 108 to 459 genomes, which was marginally increased after polishing with Illumina reads (111–464), representing the highest values of any assembly tested. Second to this were the ONT wtdgb2 assemblies that obtained 84 to 308 genomes prior to polishing with Illumina reads, and 83 to 313 after. Following this were the Illumina-based assemblies with MEGAHIT obtaining 80 to 212 viral genomes, that was further increased using binning approaches VAMB (80–216), Phables (83–218), and vRhyme (88–228). This was followed by Raven (61–213), Unicycler with ONT reads (45–140), Canu (61–135), and Unicycler with ONT and Illumina reads together (71–123). Typically, there was little difference between ONT assemblies that had been pre-processed with Filtlong or not. However, there was a substantial difference for the ONT-only Unicycler assemblies (Fig. 1a and Table S4).

Whilst ONT-based assemblies recovered the highest number of genomes with ≥50 % estimated completeness, Illumina assemblies obtained the most fully resolved viral genomes (100 % complete; Fig. 1c and Table S4). The Illumina MEGAHIT assemblies resolved 26 to 66 complete genomes, and this was increased to 33 to 81 using Phables (Fig. 1c and Table S4). The ONT-only assemblies with the most complete genomes were produced using wtdbg2, obtaining 12 to 30 complete genomes, increasing to 12 to 33 after polishing with Illumina reads. All other long-read assemblers performed poorly at recovering 100 % complete viral genomes (Fig. 1c and Table S4). Further inspection of the predicted complete genomes obtained from wtdbg2 revealed that all contained high-confidence DTRs; therefore wtdbg2 may perform better than other long-read assemblers at resolving phage termini.

As the Illumina and ONT data were sequenced from the same samples, the same phages should be present in both. Therefore, we extracted the predicted complete genomes from the pooled Phables library (*n*=81) to determine why they were not recovered in the ONT assemblies. We extracted contigs from pooled ONT assemblies that were highly like the predicted complete Phables genomes using a MASH cutoff of ≤0.05 (analogous to 95 % sequence similarity). The contigs were then compared using Mashtree.

Inspection of the resultant tree revealed that ONT assemblies frequently contained contigs that were highly like the predicted complete genomes found in the Phables assembly (Fig. S1). The ONT contigs were often of the same length as the Phables contig, however, CheckV typically predicted that the Phables contig was complete due to the presence of DTRs that were typically not resolved in the ONT assemblies. Whether these DTRs represent true DTRs or assembly artefacts is unclear. Furthermore, the median sequence depth of predicted complete Phables genomes was 99×, whereas the median for similar sequences in ONT assemblies was 15× with Filtlong and 16× without. Therefore, the lower sequencing depth of these contigs may have an impact on the quality of assembly and subsequent estimates of completeness. Furthermore, when using a completeness threshold of ≥90 % (high quality and above), more genomes are recovered by ONT assemblies, following a similar pattern to the ≥50 % completeness threshold (Fig. 1b and Table S4). When considering high-quality and above genomes, ONT assemblies produced using Flye, wtdbg2, and Raven all recovered more genomes than the Illumina assemblies (Fig. 1b and Table S4).

### Assembly quality assessment

**Fig. 2.**
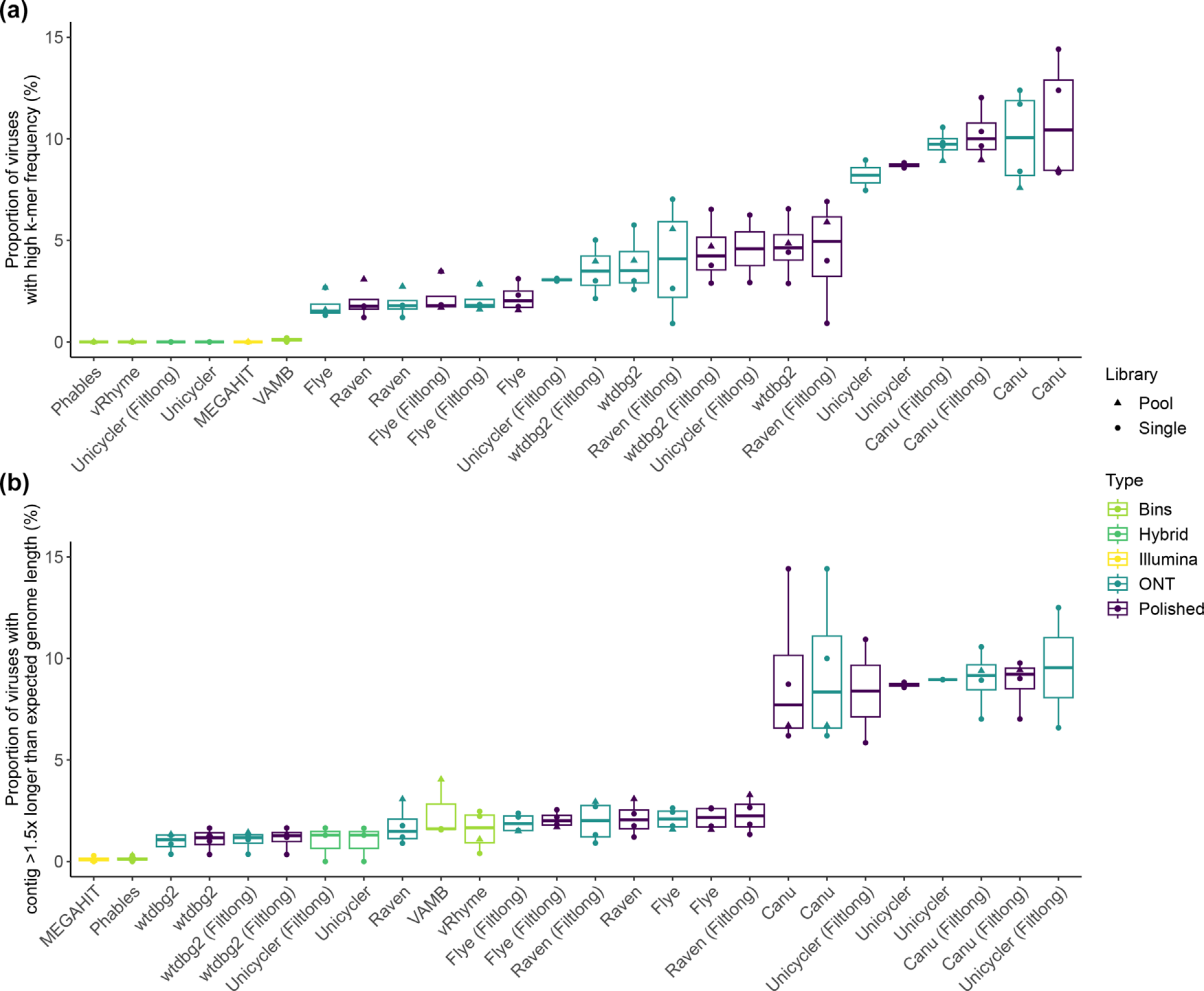
Frequency of erroneous viral genomes. The number of viral genomes per assembly with CheckV warning flags for (**a**) high k-mer frequency and (**b**) contigs exceeding 1.5× the expected genome length without being predicted as a prophage. Assemblies are sorted by median value in ascending order, showing the best performance on the left-hand side of the plot.

To assess the presence of potential errors in viral genomes, such as duplications, we used CheckV warning flags as indicators. These flags were raised either due to a high k-mer frequency or when a contig’s length exceeded 1.5× the expected genome length but was not predicted to be an integrated prophage (Fig. 2 and Table S4). Contigs with a high k-mer frequency warning likely contain a duplication, and those with a length warning could represent chimaeras.

Illumina assemblies produced using MEGAHIT had no sequences with a high k-mer frequency warning and those binned with Phables and vRhyme also had no sequences with this warning (Fig. 2a and Table S4). Binning with VAMB increased the frequency to 0.0–0.2 % of predicted viruses with the warning. Similarly, when employing direct hybrid assemblies with both Illumina and ONT reads through Unicycler, no sequences had high k-mer frequency issues (Fig. 2a and Table S4). Conversely, when focusing on ONT-based assemblies, the frequency of sequences with this warning ranged from 0.9–14.4 % per assembly, with large variation among the assemblers. The assemblies produced using Canu yielded the highest frequency (7.6–14.4 %), followed by Unicycler (2.9–9.0 %), wtdbg2 (2.1–6.6 %), Raven (0.9–7.0 %), and Flye (1.3–3.5 %; Fig. 2a and Table S4). Notably, polishing the ONT assemblies with Illumina reads typically increased the occurrence of this warning message; this may be due to the removal of errors in the ONT assembly, causing the two duplicated regions to become more similar as the errors are removed (Fig. 2a and Table S4).

Similarly, Illumina assemblies produced using MEGAHIT obtained the lowest number of sequences flagged as being >1.5× longer than the expected length (0.0–0.3 %; Fig. 2b). Those binned with Phables also obtained 0.0–0.3 % (Fig. 2b and Table S4). However, when using other binning approaches, the occurrence of this warning increased. The vRhyme assemblies ranged from 0.4–2.5 %, and VAMB from 1.6–4.0 % (Fig. 2b and Table S4). Assemblies using only ONT reads had a range of 0.4–14.4 % per assembly for this warning, with variation depending on the assembler used (Fig. 2b and Table S4). Among the ONT-only assemblies, Canu had the highest frequency (6.2–14.4 %), followed by Unicycler (5.8–12.5 %), Raven (0.9–3.3 %), Flye (1.5–2.6 %), and wtdbg2 with the lowest (0.3–1.7 %; Fig. 2b and Table S4). The levels obtained by direct hybrid assemblies produced using Unicycler with Illumina and ONT reads together were like the best performing ONT-only assemblies (0–1.7 %; Fig. 2b and Table S4). No clear pattern was observed regarding the use of Filtlong before assembly and/or the polishing process with Illumina reads after assembly in relation to this metric; for some assemblers the frequency increased and for others it decreased (Fig. 2b and Table S4).

### Differences in predicted viral diversity

To determine any differences in viral diversity recovered within each assembly, phage contigs with a CheckV completeness estimate ≥50 % (medium quality and above) were clustered to form vOTUs (95 % ANI over 85 % alignment; approximate species), which were then clustered using vConTACT2 alongside INPHARED (July 2023) to form viral clusters (VCs) that are approximately analogous to genera/subfamily-level taxonomy. We examined the number of VCs per assembly type to quantify the estimated viral diversity.

**Fig. 3. F3:**
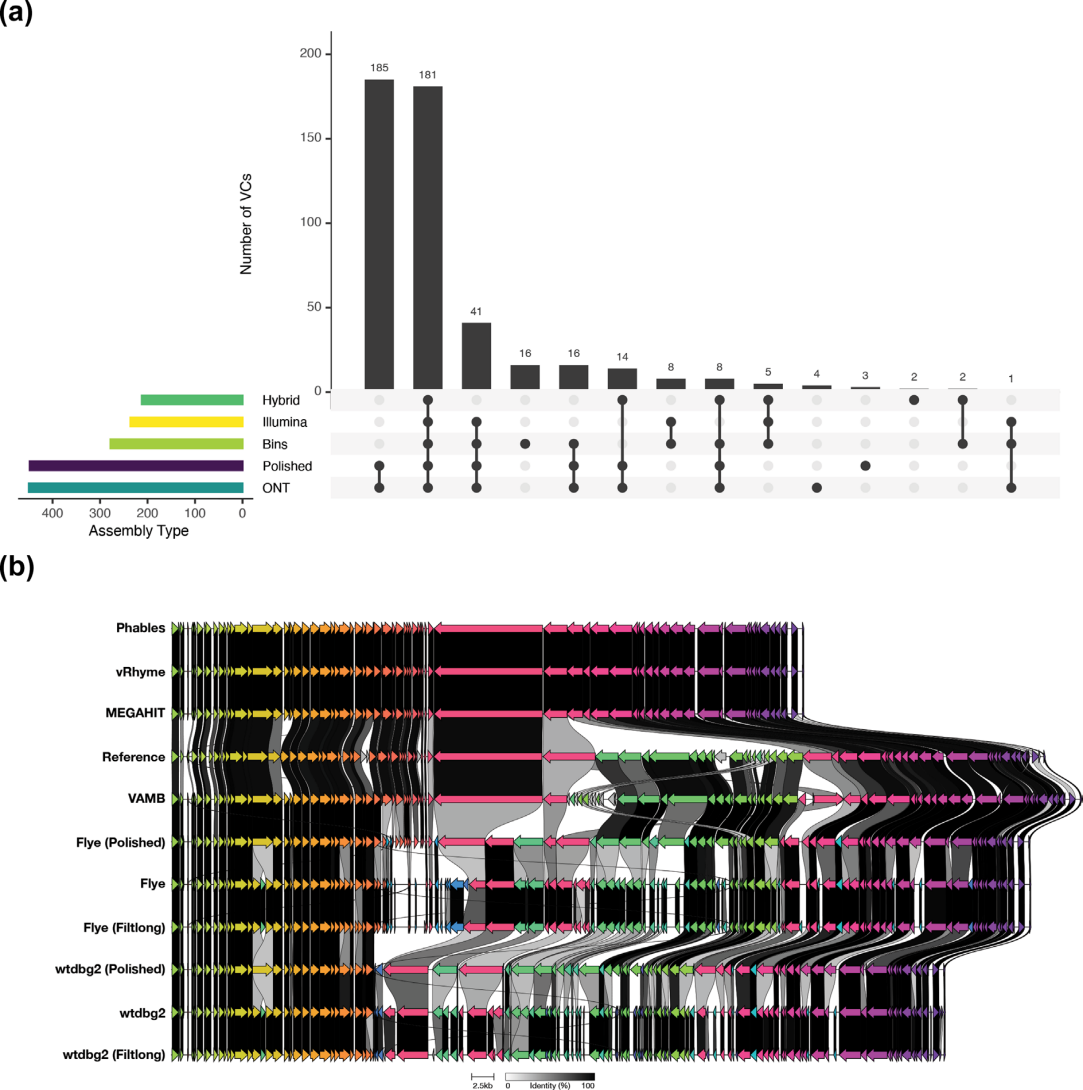
Recovery of viral clusters per assembly type. (a) The number of viral clusters recovered by each sequencing approach. (b) Comparison of synteny and genome architecture of virome sequences compared to CrAssphage LMMB (MT006214).

The ONT assemblies recovered 453 VCs across the 3 donor samples, with 185 of these being exclusive to ONT-based assemblies (Fig. 3aFi[Fig F3]g. 3a and Table S5). However, the number of VCs varied with assembler used. Flye assemblies obtained the highest number of VCs, with 97 to 349, followed by wtdbg2 (76–278), Raven (59–202), Canu (42–87), and Unicycler (39–59; and Table S5). These predictions changed very little after polishing with Illumina reads. The Illumina assemblies produced using MEGAHIT obtained 75 to 202 VCs, like Phables at 75 to 203 Fig[Fig F3]. [Fig F3]3a(Fig. 3a and Table S5). Interestingly, a slight reduction in the number of VCs recovered was observed following use of VAMB (74–171) and vRhyme (69–196; Fig. 3a). It may be that fragmented Illumina contigs derived from the same genome were clustered into separate VCs when using the Illumina MEGAHIT assemblies, and that some of these were resolved into the same genome through binning. The direct hybrid assemblies produced using Unicycler with ONT and Illumina reads together obtained the fewest VCs (74–111), and only two of these were exclusive to these assemblies (Fig. 3a and Table S5). Although most VCs could not be assigned taxonomy at the rank of family, vOTUs belonging to eight known viral families were recovered, with six of these being recovered in Illumina and ONT assemblies. However, there were two points of difference. The Illumina assemblies recovered members of *Salasmaviridae*, whereas ONT assemblies did not. The ONT assemblies recovered members of *Microviridae*, whereas Illumina assemblies did not.

To determine if the ONT-only VCs were present in the Illumina libraries but not being assembled, we mapped reads to the sequences in these VCs to determine their presence. Of the 206 VCs that were exclusive to ONT/polished/hybrid assemblies, 107 contained sequences that were present in the Illumina libraries. The Illumina reads that mapped to the ONT-only clusters were then mapped back to the vOTUs obtained from the Illumina MEGAHIT assembly. Read mapping recruited 80.3 % of reads to the MEGAHIT vOTUs, with 99 vOTUs obtaining ≥1× coverage. A large proportion of the ONT-only VCs therefore contained sequences like those in the Illumina-only assemblies. However, their forming of separate VCs is likely due to incorrect protein predictions resulting from higher error rates and erroneous stop codons.

### Recovery of CrAssphage LMMB

We noticed that several assemblies contained near-complete genomes that were highly similar to CrAssphage LMMB (MT006214). As the study of *Crassvirales* is a common component of human virome analyses, we examined these sequences in comparison to the CrAssphage LMMB to determine their completeness and synteny (Fig. 3b).Fi[Fig F3]g. 3b

The Illumina MEGAHIT sequence had high levels of nucleotide identity to that of CrAssphage LMMB with highly conserved genome architecture and synteny (Fig. 3b). However, there was a ~27 Kb segment of the genome that was missing from the MEGAHIT sequence. The use of vRhyme and Phables did not resolve this section, and their assembly was the same as that of MEGAHIT-only. However, binning with VAMB was able to recover the section that was missing from the MEGAHIT assembly, leading to the most complete genome of any assembly used (Fig. 3b). Regarding ONT assemblies, Flye was able to resolve the full length of the genome, including the ~27 Kb segment that was missing from the MEGAHIT assembly. Whilst wtdbg2 was able to resolve most of the genome, including the segment missing from MEGAHIT, a different ~11 Kb section was missing from the wtdbg2 assemblies (Fig. 3b) . While they were able to recover the full CrAssphage genome, the ONT-only assemblies contained a high number of highly fragmented ORFs (Fig. 3b). It is likely that the higher error rate associated with ONT sequencing impacted the ORF prediction on these sequences. Polishing the Flye and wtdbg2 sequences with Illumina reads led to longer ORFs that appeared to be more congruent with CrAssphage LMMB, although there was still a clear difference when compared to the reference genome and Illumina assemblies (Fig. 3b).

### Impacts of assembler on predicted protein lengths

As higher error rates are associated with the introduction of erroneous stop codons within predicted proteins, resulting in their truncation, we examined the length of predicted proteins per assembly as a proxy for error rates.

**Fig. 4.**
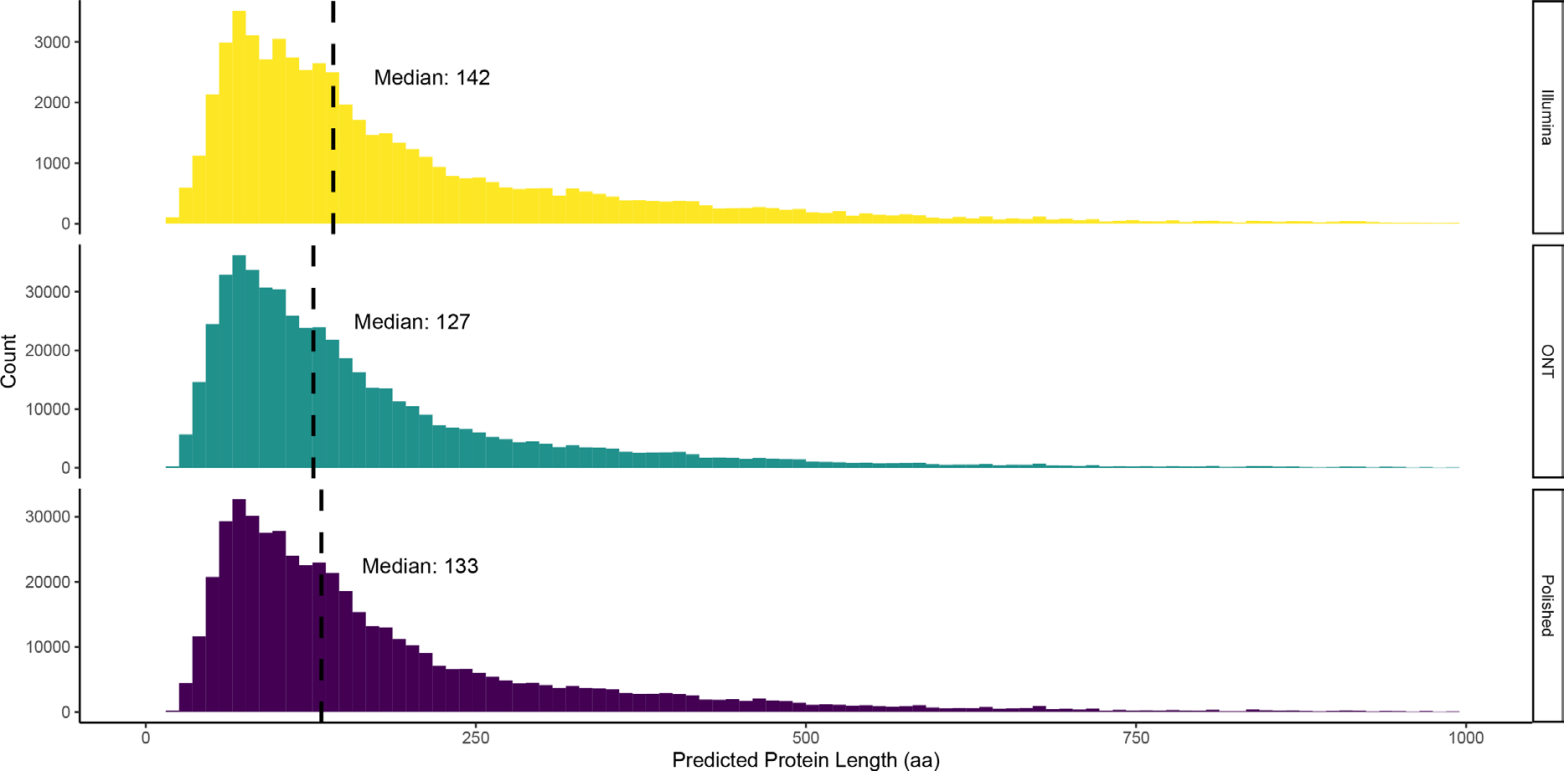
Effect of polishing on predicted protein lengths. Distribution of protein lengths (aa) with median value indicated by a dashed vertical line.

The Illumina assemblies produced using MEGAHIT had a median translated ORF length of 142 amino acids (aa), versus the ONT-based assemblies with a median length of 127 aa (Fig. 4). Polishing ONT assemblies with Illumina reads increased the median ORF length from 127 to 133 aa (Fig. 4). Although the polishing likely removed some erroneous stop codons, the ORF length was still lower than that of Illumina-only assemblies (142 aa; Fig. 4). However, the increase of median translated ORF length post-polishing did vary with assembler used. Assemblies produced using wtdbg2 increased from 120 to 128 median aa, Flye from 122 to 128 with Filtlong and from 123 to 128 without, Unicycler from 129 to 134 with Filtlong and from 136 to 143 without, Canu from 136 to 146 with Filtlong and from 134 to 145 without, and Raven from 137 to 142 with Filtlong and from 137 to 141 without Fig. S2. Therefore, polishing ONT assemblies with Illumina reads did restore ORF lengths to those comparable with Illumina-only assemblies for some assemblers tested, specifically Unicycler, Canu, and Raven.

## Discussion

The use of long-read and hybrid sequencing approaches in metagenomics is becoming more common and has been demonstrated to increase the quality and completeness of bacterial genomes [13,18]. These approaches are beginning to emerge in the field of viromics, although there are still relatively few analyses of viromes sequenced with long-read and hybrid approaches. Currently, there are few comparisons of Nanopore and Illumina sequencing for the recovery of viral genomes using mock communities [26,35]. However, there is little study into the recovery of viral genomes from complex natural samples using long-read sequencing. Here, we compared the performance of Illumina and Nanopore sequencing for the recovery of viral genomes from human faecal samples using several assembly approaches, following a workflow that is considered to be gold standard in the field. Using this approach, we are able to inform the community on issues that may arise during their viromics data analyses.

Short-read assemblers have previously been benchmarked for recovery of viral genomes from mock communities, with metaSPAdes and MEGAHIT performing best [24,75]. Whilst metaSPAdes is preferred for assembly quality, it is more computationally demanding than MEGAHIT [24,75]. Hence, we used only one short-read assembler: MEGAHIT [24,75]. For long-read sequencing, we chose several commonly used assemblers; Flye, Canu, Raven, wtdbg2, and Unicycler. Unicycler was also used for a direct hybrid assembly with combined ONT and Illumina reads. Whereas Flye, Canu, Raven, and wtdbg2 have all been developed with options for metagenome assembly, Unicycler is optimised for assembling single genomes and is therefore expected to perform less well in viromics than the other assemblers.

Determining which sequencing technology and assembler combination worked best in this work was not trivial and depends on the specific research question (Table 2). With the aim of recovering the most fully resolved viral genomes, our findings suggest Illumina is the best approach, whereas for maximising recovered viral diversity ONT is ideal (Table 2). However, previous study of Illumina sequencing for the recovery of viral genomes has uncovered false DTRs at the termini of Illumina sequences arising from repeated assembly artefacts, calling into question the validity of ‘complete’ viral genomes recovered from Illumina-only assemblies [29]. Similarly, ONT assemblies recovered far more genomes that were ≥90 % complete than Illumina, potentially due to ONT assemblies not being able to resolve DTRs at the phage termini, or perhaps through limitations in the methodology we used to determine whether genomes were complete or not.

**Table 2.** Relative performance of assembly approaches for common objectives

Viromics assembly Olympics!	Complete genomes	Maximum viral diversity	Minimise erroneous genomes	Accurate gene calls
**Gold**	Illumina+binning	ONT+Flye	Illumina	Illumina
**Silver**	Illumina	ONT+wtdbg2	Illumina+binning	ONT+polishing
**Bronze**	ONT+wtdbg2	Illumina+binning	ONT+Raven	ONT

Conversely, the increase in predicted viral diversity from ONT sequencing is not necessarily a clear benefit, as further analysis of the data brings the reliability of the predictions into question. We found that much of the increased diversity in the ONT data is likely the result of erroneous protein predictions, leading to incorrect clustering of contigs using gene-based approaches. This finding is consistent with a previous comparison in which long-read assemblies were shown to vastly overestimate viral diversity within a mock community analysis [35]. Furthermore, there was also the associated cost of higher frequencies of erroneous genomes and higher error rates. However, there are reports of virus genomes not being recovered with Illumina sequencing, whereas ONT could potentially provide a solution, even if more error prone [76,77]. It should be acknowledged that the Illumina assemblies had a higher sequencing depth than the ONT assemblies, which may have influenced the quality of the assemblies and predictions of genome completeness.

Therefore, taken altogether, we suggest that if only one sequencing technology were used for a virome analysis, Illumina should be preferred to ONT. Although the ONT assemblies performed favourably in recovering viral diversity, the input requirements and need for amplification are still the largest barrier for virome sequencing. However, long reads are not without their benefits. The use of hybrid approaches that combine Illumina sequencing with ONT may best capture viral diversity within a sample and overcome issues associated with amplification, although this increases costs in using more than one sequencing technology [25,26, 35].

The continual development and improvement of ONT flow cells and assembly algorithms will improve the quality of assemblies. To date, there is relatively little optimisation of ONT sequencing specifically for viromics. For example, it is well documented that bacteriophages often have heavily modified DNA, and this may impact on the accurate basecalling of their genomes [78,81]. It was also previously reported that too much sequence depth is detrimental for ONT virome assembly [35], although downsampling prior to assembly will remove genomes of lower abundance. Furthermore, there are clear differences in ONT assemblers, with no single assembler performing best in all metrics. In bacterial genomics, the use of multiple assemblers can generate more trustworthy consensus sequences in the form of Trycycler [82]. There is currently no such approach for metagenomes and viromes. Similarly, our analyses included binning algorithms for Illumina data but not for ONT data. The Illumina-focused binning algorithms (Phables, VAMB, and vRhyme) have been optimised for viral metagenomes [46,48]. More recently, another approach for resolving Illumina-based assembly graphs, COBRA, was shown to increase the completeness and contiguity of viral genomes assembled in metagenomes [83]. Whilst binning algorithms for ONT data are emerging, we are not aware of any that have been validated using viral metagenomic data [84]. Likewise, approaches to mitigate the effect of frameshift errors have been developed for ONT assemblies, although they cannot be readily applied to virome analyses due to the limitations of existing reference databases [85]. To accurately uncover more viral diversity from natural samples, there is a clear need for assembly algorithms and library preparation methods that are optimised for viral metagenomics.

On the laboratory side, the development of the VirION2 protocol, which included optimisations in the choice of polymerase, PCR cycles, and DNA shearing, has led to increased read lengths and reduced the input DNA requirement to 1 ng [26]. Unfortunately, the laboratory work for this study was initiated before the VirION2 protocol was published and we therefore could not incorporate some of the optimisations. Additionally, other emerging long-read technologies could be promising for viral metagenomics. Notably, PacBio HiFi sequencing is able to improve the completeness of bacterial MAGs and obtain far lower error rates than ONT sequencing [86], with further improvements being made in specialist HiFi read assembly algorithms [13,18].

Even with improvements that allowed for increased genome recovery from viral metagenomes, there are still large biases in the viruses recovered. This study, and the overwhelming majority of virome studies, focus exclusively on the DNA fraction and this is further biased towards dsDNA viruses. Recent mining of global metatranscriptomes has expanded currently known RNA viral diversity and suggests that RNA viruses may be a critical but currently neglected component of the global virome [87]. There are virome protocols that include cDNA synthesis for the sequencing of RNA alongside DNA [88], but the resulting fragments may be too short to take advantage of long reads. Whilst direct RNA sequencing with ONT is available, the input requirements are prohibitive for most virome studies. There are still many technical challenges to capturing true viral diversity within an environmental sample.

## Conclusions

The use of viral metagenomics has accelerated our understanding of viral diversity within a plethora of environments, although much viral diversity remains unseen and continual improvements to library preparation methods, sequencing technologies, and assembly algorithms are needed to understand true viral diversity. Illumina-based approaches may still offer the current gold standard for viromics, and the use of viral-specific binning algorithms, such as Phables, may aid the recovery of viral genomes. The addition of long reads uncovers additional viral diversity, although stringent quality control should be used to minimise the occurrence of erroneous viral genomes. As the choice of sequencing technology and assembly approach will impact on the observed viral diversity, these methodological choices should always be considered in the interpretation of results.

## supplementary material

10.1099/mgen.0.001236 Uncited Fig. S1.

10.1099/mgen.0.001236 Uncited Table S1.

## Abbreviations

ANI: average nucleotide identity
LASL: linker-amplified shotgun library
MAG: metagenome-assembled genome
MDA: multiple displacement amplification
MIUViG: Minimum Information about an Uncultivated Virus Genome
ONT: Oxford Nanopore Technologies
ORF: open reading frame
PacBio: Pacific bioscience
VC: viral cluster
vOTU: viral operational taxonomic unit
