# Supplementary material for: Nanopore and Illumina sequencing reveal different viral populations from human gut samples
Source: Microb Genom. 2024 Apr 29;10(4):001236. doi: 10.1099/mgen.0.001236 (PMC11092197; doi:10.1099/mgen.0.001236)
Supplement: Uncited Fig. S1. [file mgen-10-01236-s001.pdf]

Supplementary Figures

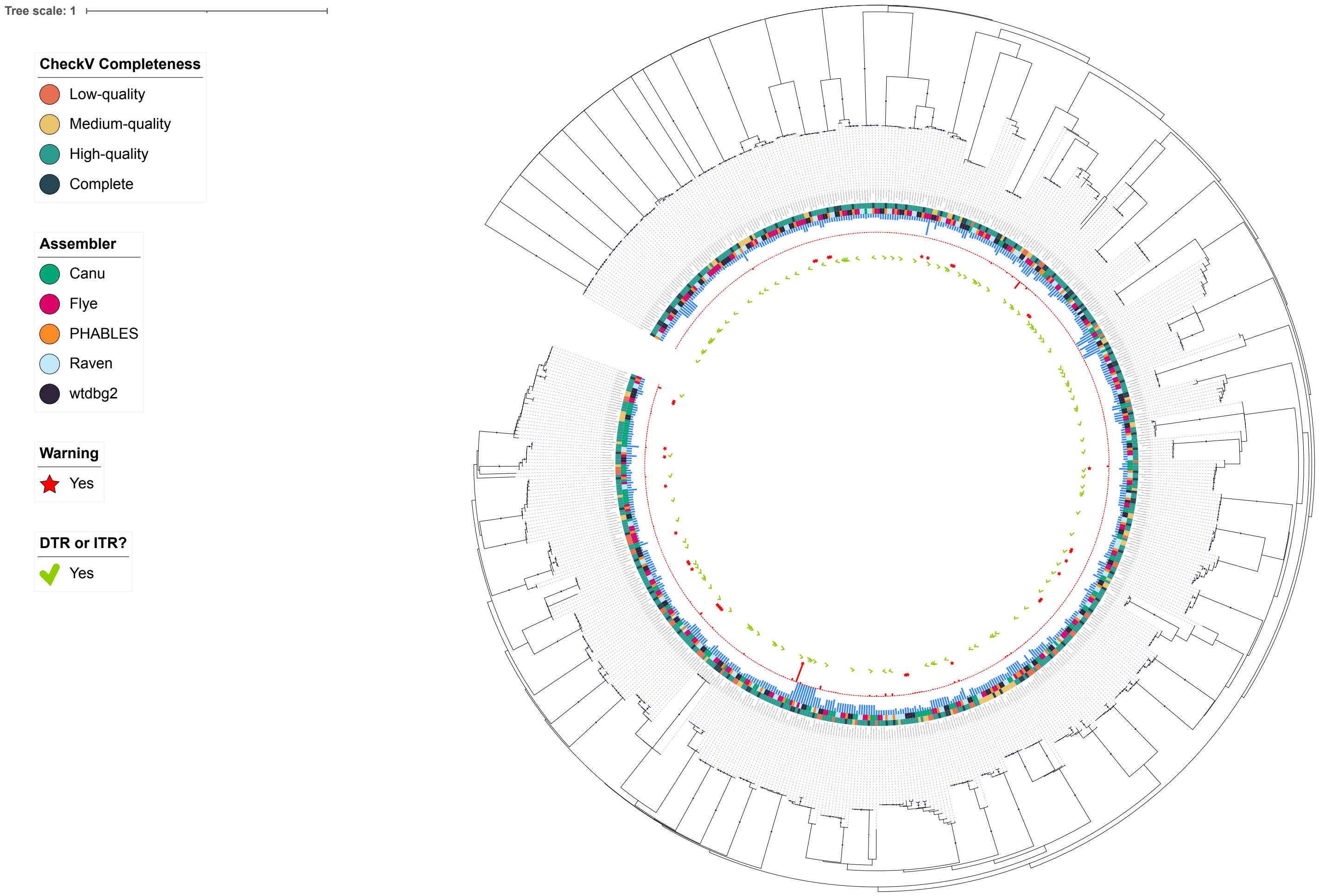

Supplementary Figure 1. MASHTREE of predicted complete genomes from the pooled Phables assembly, alongside closest matching sequences from pooled ONT assemblies. Outermost ring (closest to nodes) indicates CheckV completeness score, inner ring indicates assembler used. Blue bars indicate contig length and red bars indicate sequence depth. Red stars indicate a CheckV warning flag for either contig length or k-mer frequency. Green ticks indicate completeness was determined by presence of DTRs or ITRs. Bootstrap support is shown with the black circle, with a minimum value of 70 displayed.

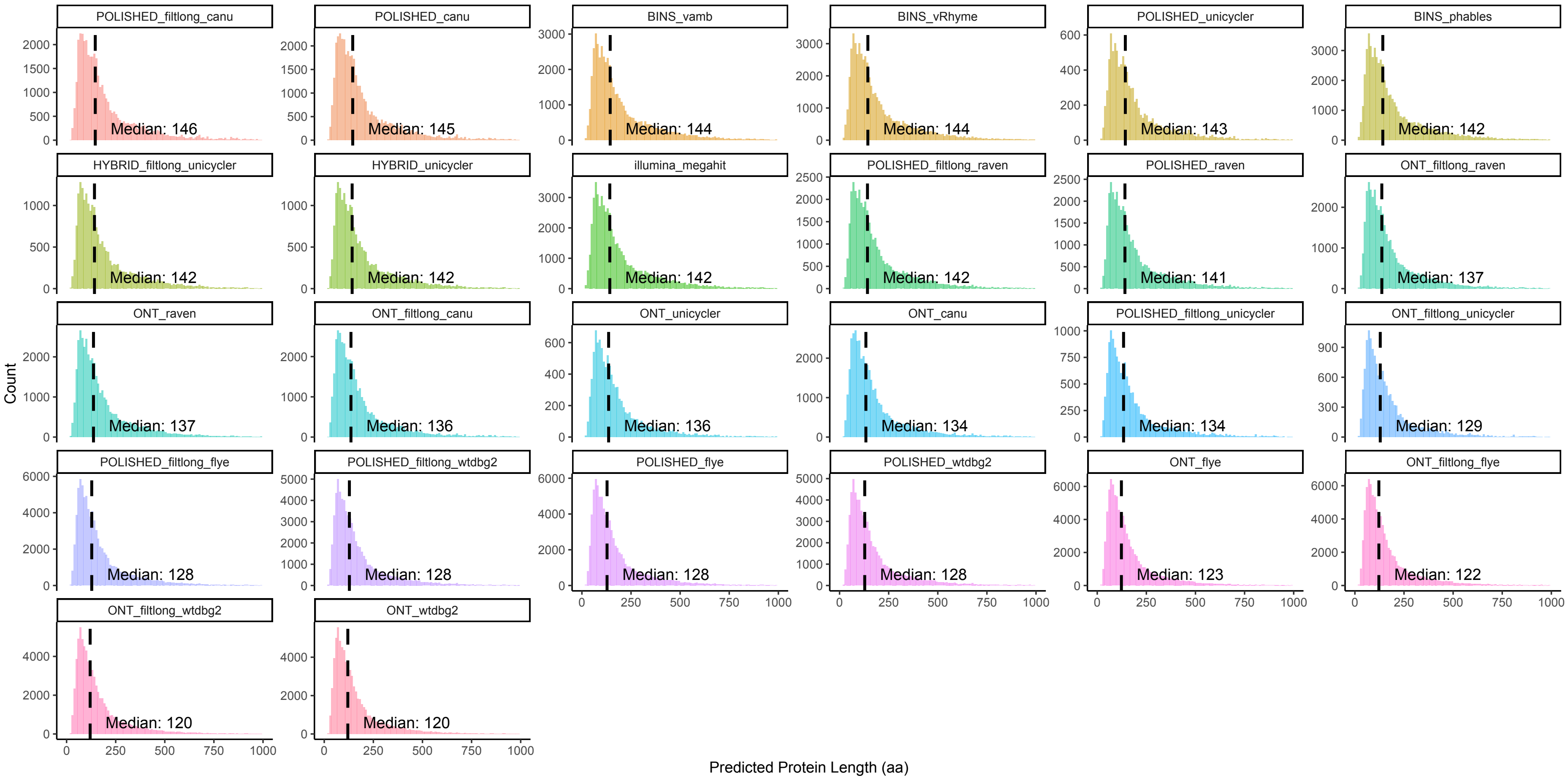

POLISHED_filtlong_canu
Median: 146
POLISHED_canu
Median: 145
BINS_vamb
Median: 144
BINS_vRhyme
Median: 144
POLISHED_unicycler
Median: 143
BINS_phables
Median: 142
HYBRID_filtlong_unicycler
Median: 142
HYBRID_unicycler
Median: 142
illumina_megahit
Median: 142
POLISHED_filtlong_raven
Median: 142
POLISHED_raven
Median: 141
ONT_filtlong_raven
Median: 137
ONT_raven
Median: 137
ONT_filtlong_canu
Median: 136
ONT_unicycler
Median: 136
ONT_canu
Median: 134
POLISHED_filtlong_unicycler
Median: 134
ONT_filtlong_unicycler
Median: 129
POLISHED_filtlong_flye
Median: 128
POLISHED_filtlong_wtdbg2
Median: 128
POLISHED_flye
Median: 128
POLISHED_wtdbg2
Median: 128
ONT_flye
Median: 123
ONT_filtlong_flye
Median: 122
ONT_filtlong_wtdbg2
Median: 120
ONT_wtdbg2
Median: 120
Count
Predicted Protein Length (aa)

Supplementary Figure 2. Histograms of predicted protein length for ONT assemblies, with median indicated by the dashed line. Plots are in descending order of median value.
